# Directed Connectivity Analysis of the Neuro-Cardio- and Respiratory Systems Reveals Novel Biomarkers of Susceptibility to SUDEP

**DOI:** 10.1109/OJEMB.2020.3036544

**Published:** 2020-11-06

**Authors:** T. Noah Hutson, Farnaz Rezaei, Nicole M. Gautier, Jagadeeswaran Indumathy, Edward Glasscock, Leonidas Iasemidis

**Affiliations:** ^1^ Department of Biomedical EngineeringLouisiana Tech University 5160 Ruston LA 71272 USA; ^2^ Department of Mathematics and StatisticsLouisiana Tech University 5160 Ruston LA 71272 USA; ^3^ Department of Cellular Biology and AnatomyLouisiana State University Health Sciences Center 23346 Shreveport LA 71130 USA; ^4^ Department of PhysiologyJawaharlal Institute of Postgraduate Medical Education and Research 29988 Puducherry India; ^5^ Department of Biological SciencesSouthern Methodist University 2765 Dallas TX 75275 USA; ^6^ Department of Biomedical EngineeringLouisiana Tech University 5160 Ruston LA 71272 USA; ^7^ Center for Biomedical Engineering and Rehabilitation ScienceLouisiana Tech University 5160 Ruston LA 71272 USA

**Keywords:** Epilepsy, SUDEP, Brain-Heart-Lungs, Functional Network Connectivity, Dynamics and Biomarkers

## Abstract

Sudden unexpected death in epilepsy (SUDEP) is the leading cause of epilepsy-related mortality and its pathophysiological mechanisms remain unknown. *Goal:* We set to record and analyze for the first time concurrent electroencephalographic (EEG), electrocardiographic (ECG), and unrestrained whole-body plethysmographic (Pleth) signals from control (WT - wild type) and SUDEP-prone mice (KO- knockout Kcna1 animal model). *Methods:* Employing multivariate autoregressive models (MVAR) we measured all tri-organ effective directional interactions by the generalized partial directed coherence (GPDC) in the frequency domain over time (hours). *Results:* When compared to the control (WT) animals, the SUDEP-prone (KO) animals exhibited (p < 0.001) reduced afferent and efferent interactions between the heart and the brain over the full frequency spectrum (0-200Hz), enhanced efferent interactions from the brain to the lungs and from the heart to the lungs at high (>90 Hz) frequencies (especially during periods with seizure activity), and decreased feedback from the lungs to the brain at low (<40 Hz) frequencies. *Conclusions:* These results show that impairment in the afferent and efferent pathways in the holistic neuro-cardio-respiratory network could lead to SUDEP, and effective connectivity measures and their dynamics could serve as novel biomarkers of susceptibility to SUDEP and seizures respectively.

## I. Introduction

Sudden unexpected death in epilepsy (SUDEP), the leading cause of death in epilepsy with an incidence rate of roughly 0.5% of the epilepsy patient population per year, has only recently begun to be addressed [1]. Although the underlying pathophysiological mechanisms are still unknown, several studies suggest that impaired cardiorespiratory function during seizures (ictal periods) or immediately after seizures (post-ictal periods) may play a significant role in the occurrence of SUDEP [2], [3]. In a study of electroencephalography (EEG), oxygen saturation levels, thoracoabdominal excursions, and electro-cardiography (ECG) with epilepsy patients, central apneas occurred during generalized convulsive seizures and post-ictal periods [4]. In a larger cohort of patients with focal epilepsy, central apneas were reported during and after 37% of seizures [5]. In a heart-monitoring study of implantable loop recorders in epilepsy patients over 24 months, peri-ictal cardiac arrhythmias were observed. Ictal bradycardia was rare, but patients exhibiting this activity were clinically characterized as having the highest risk for SUDEP [6]. Taken together, seizure-associated apneas and arrhythmias in clinical studies indicate that seizures may induce a lapse in the functional connectivity between the brain and cardiorespiratory systems leading to impaired neuronal control of breathing and cardiac function that could increase risk of SUDEP.

The cardiorespiratory system consists of strongly coupled and interdependent organs and tissues that deliver oxygen and other nutrients to the rest of the body [7], [8]. The tight inner connectivity of this system is also in part due to its connections with the central nervous system control centers for cardiac and respiratory activity. Strong afferent pathways from the cardio-respiratory system project to vital control centers in the brain relaying electrochemical and barometric information [9]–[11]. In epilepsy, the electrochemical coupling among the brain, heart and lungs can be altered by inherited abnormalities, as well as developed ones such as acquired channelopathies in cardiac tissue [12]. Seizures can impact the electrochemical coupling through the vagus and glossopharyngeal nerves, as well as metabolic and hormonal coupling linked by the vasculature [13]. In a study of cardiac stress biomarkers in epilepsy patients, significant increases (up to two-fold) in the levels of cardiac stress proteins (cardiac troponin I [cTNI]; high-sensitive troponin T [hsTNT]) were identified within 30 minutes following seizures [14]. Despite advances in identifying potential SUDEP mechanisms, validated and quantitative risk factors and biomarkers of SUDEP susceptibility remain undetermined. One reason is the inherent genotypic and phenotypic complexity of epilepsy and its varying impact on the cardio-respiratory system [3], [15]. Another factor is the inadequate (e.g., not concurrent) recording and analysis of the recorded signals from the neuro-cardio-respiratory systems, often focusing on one single node (i.e., heart, lungs or brain) or between two nodes in the network at a time.

Biosignal analysis for measuring both direct and indirect interactions among brain, heart and lungs has been attempted in the past. In 2005, a non-linear measure of cardiorespiratory coupling was introduced to capture really low-frequency stochastic oscillatory behavior between heart and lung activity [16]. In 2007, similar investigations in two groups of rats undergoing two different methods of anesthesia each revealed frequency dependent coupling of the brain, heart, and lungs, with an underlying driving relationship from the lungs to the heart which was amplified by anesthetic agents [17]. In 2016, analysis of anesthetized human subjects revealed significant changes in low frequency coupling of the neuro-cardio and cardio-respiratory systems following anesthesia [18]. Interactions between the heart and brain have also been investigated in epilepsy. Statistically significant pre-ictal increases and post-ictal drops in brain-heart connections were detected in children with temporal lobe epilepsy (TLE) through non-linear information measures from simultaneously recorded EEG and ECG. Employing empirical mode decomposition (EMD) on EEG and ECG in 10- and 20-minute peri-ictal recordings, significant drops in the brain-heart interactions were noticed immediately prior to and during seizures [19]–[21]. However, despite significant research in this area, the neuro-cardio-respiratory system as a unified and directed network in epilepsy has been largely ignored and unexplored.

Due to lack of reliable biomarkers, and since SUDEP occurs suddenly and unexpectedly, it is very difficult to reliably and prospectively identify patients at risk and then monitor them in the long term. Thus, SUDEP investigations in humans tend to be hindered by smaller sample sizes that prevent high statistical power. Preclinical animal models can overcome this limitation and provide a suitable system to identify novel biomarkers for future validation in focused patient cohorts. One of the most prominent mammalian SUDEP models currently in use is the *Kcna1* gene knockout (KO) mouse. The *Kcna1* gene encodes axonal membrane voltage-gated Kv1.1 potassium channel $\alpha $-subunits that reduce neuronal excitability by repolarizing membrane potential following an action potential and preventing repetitive firing [22], [23]. KO mice lacking Kv1.1 channels due to deletion of the *Kcna1* gene (*Kcna1^–/–^*) are frequently utilized to investigate genetic and pathophysiological mechanisms underlying SUDEP since about 75% of animals exhibit seizure-related sudden death before the age of 2 months [24]. In addition, they exhibit similarities with multiple SUDEP risk factors and terminal neuro-cardiac patterns observed in humans including: (i) frequent seizures, (ii) generalized tonic–clonic seizures, (iii) early onset epilepsy; (iv) long duration of seizures; (v) seizure-evoked bradycardia and asystole progressing to cardiac arrest; and (vi) seizure-evoked breathing dysfunction [25]–[28]. Our group has reported in the past that KO mice also exhibit abnormal brain-heart dynamics characterized by dissociation of EEG and ECG biosignals, as measured by a decrease in the phi coefficient, a measure of binary correlation between brain (EEG) and heart (ECG) activity [29]. We have also reported that during interictal periods, KO mice exhibit additional evidence of brain-mediated cardiorespiratory dysfunction including a 5-fold increase in vagally-driven atrioventricular cardiac conduction blocks; increased heart rate variability; a 3-fold increase in respiratory variability; and a nearly complete absence of post-sigh-apneas [30]. However, quantitative assessment of the global relationship between brain, cardiac, and respiratory activities in KO mice has never been performed. To this goal, in this work, we performed comparative analysis of concurrent brain-heart-lung signals from wild type (WT) and KO *(SUDEP*-prone) mice to identify novel biomarkers associated with increased SUDEP risk. In addition to providing advanced warning for susceptibility to SUDEP and possibly to status epilepticus (SE), these findings could be found useful in identifying seizure severity, as well as enabling development of reliable biomarkers for evaluating and monitoring the efficacy of different epilepsy treatments.

## II. Materials and Methods

### A. SUDEP-Prone Subjects

The *Kcna1* knockout (KO) allele (*Kcna1*^–/–^) was generated by targeted deletion of the entire open reading frame of the *Kcna1* gene on mouse chromosome 6 leading to complete loss of Kv1.1 function, as previously described [25]. As a result, KO mice exhibit spontaneous seizures and premature death in up to about 75% of animals. The wild type (WT) and KO mice participated in this study were of a mixed genetic background (50% Black Swiss (Tac:N:NIHS-BC) and 50% C57BL/6J) as previously described in [29]. Mice were housed at ∼22 °C, fed ad libitum, and submitted to a 12-h light/dark cycle. All procedures were performed in accordance with the guidelines of the National Institutes of Health (NIH), as approved by the Institutional Animal Care and Use Committee of the Louisiana State University Health Sciences Center-Shreveport.

### B. Concurrent EEG-ECG-Pleth Recordings

**Fig. 1.**
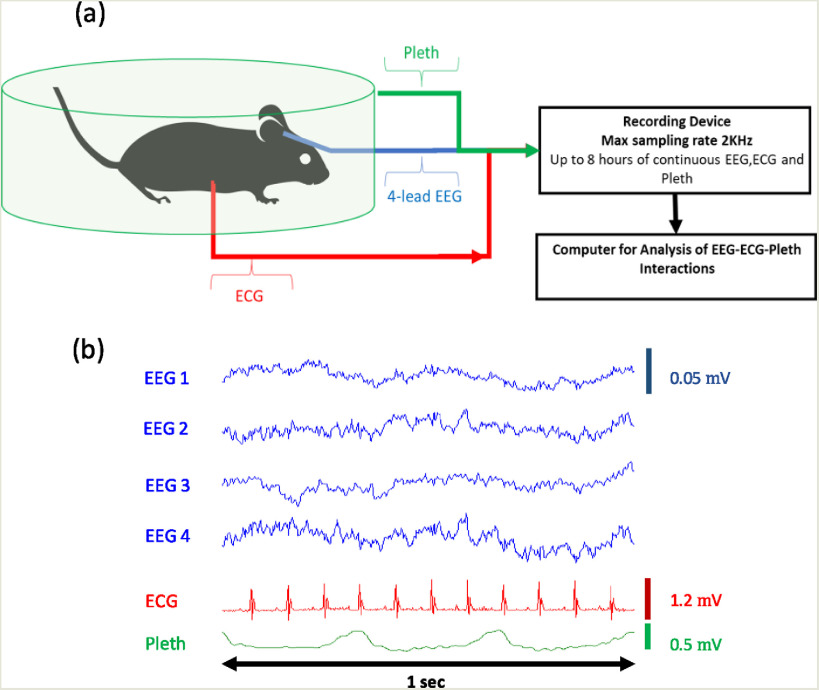
(a) Schematic diagram of the recording electrodes in a mouse, including the unrestrained whole-body plethysmography chamber used to measure pressure changes from breathing that are converted into respiratory waveforms. (b) Exemplary 1-s concurrent recordings of EEG (4-channel), ECG and Pleth data from a wild type (WT) mouse. EEG1, EEG2, EEG3 and EEG4 were recorded from the animal's left temporal cortex, right temporal cortex, left parietal cortex and right parietal cortex, respectively (see text for details).

**WT (n=7) and KO mice (n=8)** of both sexes (ages 4–6 weeks) were surgically implanted with bilateral silver wire electrodes (0.005-inch diameter) attached to a microminiature connector for recording in a tethered configuration, as described previously in [31], [32]. Avertin (0.02 mL/g, i.p.) was prepared by mixing 250 mg 2,2,2-tribromoethanol (Sigma-Aldrich) with 155 μL 2-methyl-2-butanol (Sigma-Aldrich) dissolved in 19.75 mL double-distilled water heated to 40 °C, followed by filter-sterilization prior to use. **Four EEG electrodes** (four channels) were inserted into the subdural space through cranial burr holes overlying left and right temporal and parietal cortices, and two electrodes were inserted above the left and right frontal cortices for a reference and ground, respectively. **For ECG, two thoracic electrodes** (one channel) were tunneled subcutaneously on either side and sutured in place to record cardiac activity. Mice were allowed to recover for 3 days and then placed in an unrestrained **whole-body plethysmography (Pleth)** chamber (Data Sciences International; St Paul, MN) to record respiratory waveforms (see Figure 1(a)). The lid of the Pleth chamber was modified to accommodate wires for recording EEG-ECG in a tethered configuration [33]. Following a 45-min acclimatization period in the chamber, video and EEG-ECG-Pleth were simultaneously recorded in up to 8-h sessions during the daytime (i.e., between 6:00 am and 6:00 pm) using Ponemah data acquisition and analysis software (Data Sciences International, St. Paul, MN, USA). The sampling rates were 500 Hz for Pleth and EEG, and 2 kHz for ECG. A schematic diagram of the experimental setup for recording concurrent EEG-ECG-Pleth signals, in addition to exemplary EEG-ECG-Pleth data down-sampled to 500 Hz, are shown in Figure 1(b). Seizures in KO mice were identified by visual inspection of video and EEG as high-amplitude, rhythmic electrographic discharges lasting ≥5 s.

### C. Connectivity Analysis of EEG-ECG-Pleth Signals

Bivariate analysis is likely to give misleading connectivity values between two parts of a system in cases of existing global interactions among more than two different parts of the system. Therefore, appropriate analytical tools using multivariate analysis should be utilized to correctly measure bivariate interactions in such systems. Multivariate autoregressive (MVAR) modeling over concurrent short-time epochs (windows), each from multiple time series, is recommended for network connectivity analysis assuming that these time series (signals) are recorded from different parts of a multi-dimensional, linear and wide-sense stationary system. MVAR modelling reduces computational cost and allows exploratory insight into a system. For each time window, the estimated array of MVAR model coefficients can then be further analyzed in the frequency domain and, depending on different types of normalization utilized, provide frequency-specific measures of directional functional connectivity (e.g., inflow, outflow, effective inflow, effective outflow etc.) between the nodes of the assumed network configuration of the system. One such directed connectivity measure is the Generalized Partial Directed Coherence (GPDC) that provides an accurate measure of effective direct inflow from one node to another at particular frequencies [34]. We have successfully employed GPDC in network analyses of intracranial EEG (iEEG) [32]–[36], and magnetoencephalographic (MEG) recordings [37] from patients with focal epilepsy for localization of their epileptogenic focus, as well as assessment of the dynamics of brain's network connections en route to status epilepticus [38]. GPDC exhibits certain advantages over other linear measures of causal interactions in heavily interconnected networks. Compared to more traditional tests of Granger Causality, such as Directed Coherence (DC) and Directed Transfer Function (DTF) and its variants [35], [39] GPDC allows for more accurate identification of direct (not indirect) network activity through employment of partial coherences [34]. Another key feature of GPDC is that the employed normalization allows the measure to be scale independent, thus paving the way to assess the effective connectivity between signals of possibly vastly different magnitudes, like signals from different organs. The digitized ECG were first low-pass filtered to 200Hz using a 4th order Butterworth digital filter and then down-sampled from 2 KHz to 500 Hz to match the 500 Hz sampling rate of the EEG and Pleth signals. We then fit the multivariate X(t) data (4 EEG, 1 ECG and 1 Pleth channels) by MVAR and estimated all statistically significant (P < 0.05) GPDC connectivities between the available time series in the frequency domain, from 1 to 200 Hz. In particular, a D=6 dimensional MVAR model was fitted to consecutive and non-overlapping running windows of 10 s over 4-hour EEG-ECG-Pleth recordings from the 6 available channels using a model order p=7 per WT and KO animal. The model order of 7, and window length of 10 s, for each data segment follows our line of research with scalp and intracranial EEG (iEEG) on seizure prediction and control [40], [41], [50]–[59], [42], [60]–[64], [43]–[49]. An epoch (window length) of 10 s (5000 data points x 6 channels= 30,000 data points) is enough for a confident estimation of the 7x6x6= 252 MVAR parameters as we are using more than 100x as many data points as we have parameters to fit. A short description of the methodology, including evidence that convergence of the main results of this study is achieved with model orders above 5, and the accompanied statistical significance derivations are provided in the Supplement.

## III. Results

### A. Neuro-Cardio-Respiratory Connectivity in WT and KO Animals

**Fig. 2.**
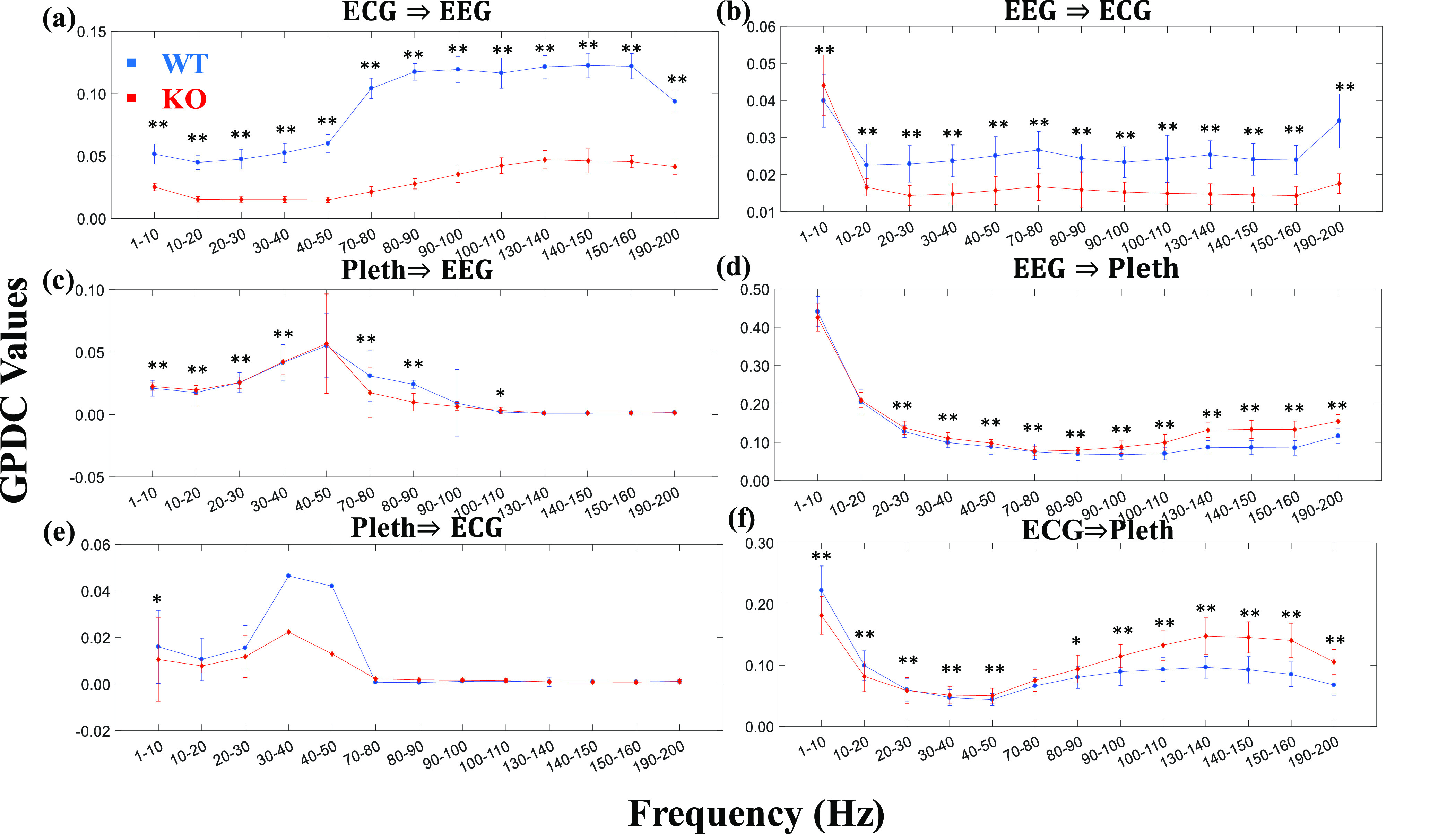
Mean  ± SEM of statistically significant directional connectivities between organs (P < 0.05) over the 4-h EEG-ECG-Pleth recordings in the frequency domain (10 Hz consecutive bands from 1 to 200 Hz; bands with harmonics of 60Hz power noise having been excluded) for the WT (blue) and SUDEP-prone KO (red) genotypes. (a) Heart ⇒ Brain, (b) Brain ⇒ Heart; (c) Lungs ⇒ Brain, (d) Brain ⇒ Lungs; (e) Lungs ⇒ Heart and (f) Heart ⇒ Lungs. Interactions for which the differences between their median values across the two genotypes are statistically not equal as estimated with the non-parametric Mann-Whitney-Wilcoxon rank sum test are indicated by (*) for P-value <0.05, and by (**) for P-value<0.001.

We first sought to uncover general trends in neuro-cardio-respiratory connectivity per interaction between organs within each genotype and then across the two genotypes (WT and KO). Towards this goal, we integrated the statistically significant GPDC (ssGPDC) values between any of the three organs (brain, heart, lungs) across time (over all 10 s windows) per frequency band for all animals of each genotype. Thus, the profiles of the mean and the standard error of the mean (SEM) of the ssGPDC values for both genotypes were calculated per frequency band for each of the six inter-organ interactions. Profiles of the ssGPDC values for both genotypes and each directional interaction are shown in the frequency domain in Figure 2 with a zero value assigned to the mean of interactions in frequency bands where there were no statistically significant interactions (p>0.05). P-values used to populate this figure can be found in the supplementary materials on **Table S1.**

Several observations from Fig. 2 are noteworthy. We classify them into the following three categories:

A) **Trends in connectivity as function of frequency for each individual interaction between organs:** The ***Heart***$ \Rightarrow $***Brain*** interactions were higher in the high frequency band (above 40 Hz) than in the low frequency band (below 40 Hz), while the ***Brain***$ \Rightarrow $***Heart*** interactions were relatively constant within the broad frequency band of 20-180 Hz (Fig. 2(a) and (b)); the ***Lungs***$ \Rightarrow $***Brain*** interactions were highest around 40 Hz, while the ***Brain***$ \Rightarrow $***Lungs*** interactions were highest around 10 Hz with a tendency towards high values again above 100 Hz (Fig. 2(c) and (d)); the ***Lungs***$ \Rightarrow $***Heart*** interactions were also highest around 40 Hz, while ***Heart***$ \Rightarrow $***Lungs*** interactions had a trend similar to the one for the ***Brain***$ \Rightarrow $***Lungs*** interactions (Fig. 2(e) and (f)). All these trends were qualitatively similar for both WT and KO mice.
B) **Relative strength of each interaction with respect to the other interactions:** The observed relative strengths of the interactions overall (i.e., across the full spectrum) were similar in both WT and KO mice, with the exception of ***Heart*** $\Leftrightarrow$ ***Brain*** interactions. The order of interaction pairs with respect to their magnitude was: ***[Brain***$ \Rightarrow $***Lungs] >* *[Heart***$ \Rightarrow $***Lungs]* *⋍* *[Heart***$ \Rightarrow $***Brain] > rest of interactions.*** Statistical comparison between all possible interaction pairs is provided in **Table S2**.
C) **Comparison of each inter-organ interaction between KO and WT animals:** Across genotypes, several important differences per interaction between organs emerged. The most prominent difference was found to be in the ***Heart*** $\Leftrightarrow$ ***Brain*** (bi-directional) interactions where the KO mice exhibited reduced functional connectivity in both afferent and efferent directions and almost over the full frequency band (20-200 Hz) (Fig. 2 (a) and (b)) compared to WT mice**.** The next prominent differences in organ interactions across genotypes were exhibited in narrower frequency bandwidths. For example, the efferent pathways from the brain and heart to the lungs **(*Brain***$ \Rightarrow $***Lungs*** and ***Heart***$ \Rightarrow $***Lungs)*** were found elevated in KO vs. WT mice at frequencies above 100 Hz (Fig. 2 (d) and (f))**.** On the contrary, the afferent pathways to the brain and heart from the lungs **(*Lungs***$ \Rightarrow $***Brain*** and ***Lungs***$ \Rightarrow $***Heart)*** were found lower in KO vs. WT mice within narrow frequency bands below 100 Hz (Fig. 2(c) and (e)).

**Fig. 3.**
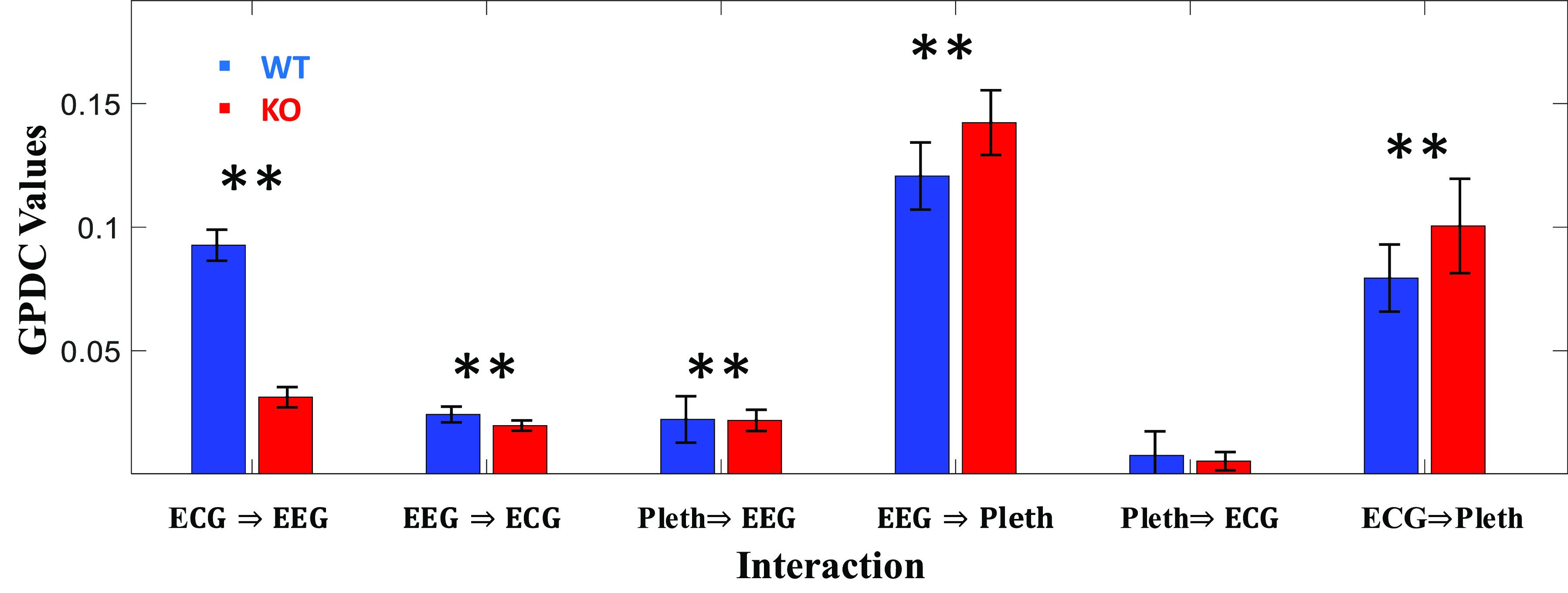
Mean  ± SEM of the averaged over the full frequency domain (0-200Hz) directional interactions between brain, heart and lungs in WT (blue) and SUDEP-prone KO (red) mice. (*) indicates the interactions for which the differences in their median values between the two genotypes are statistically not equal (P-value <0.001), as estimated with the non-parametric Mann-Whitney-Wilcoxon rank sum test.

The ***Heart*** $ \Rightarrow $***Brain*** impairment in KO vs. WT animals observed in Fig. 2(a) is still very prominent if we average the ssGPDC values over the full spectrum from 0 to 200 Hz. Figure 3 shows cumulatively (averaged over all frequencies and all available 10-s windows) the strength of all ssGPDC interactions per genotype.

**Fig. 4.**
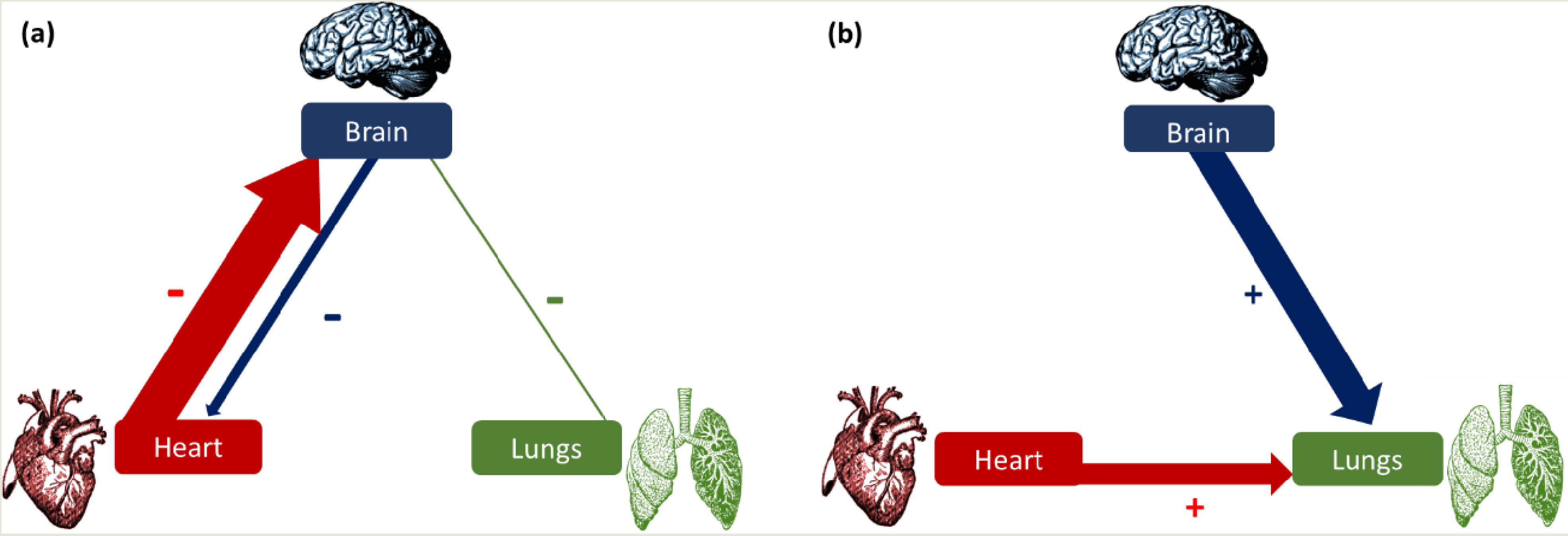
Schematic representation of the statistically significant (a) decreased and (b) increased neuro-cardio-respiratory network interactions of the KO compared to WT mice averaged over frequencies of 1-200 Hz. The thickness of arrows corresponds to the magnitude, and the (+) or (-) sign above the arrows to the signs of the difference of the ssGPDC values of KO from the ones of WT animals for each interaction.

Although it is visually less obvious in Fig. 3 than in Fig. 2, the efferent interaction from ***Brain*** $ \Rightarrow $***Heart*** is also significantly reduced in its median value in KO compared to the one in WT animals. The inter-organ interactions averaged over all frequencies that are statistically significant different between the two genotypes are schematically depicted in Figure 4. The representation in Fig. 4, derived from Fig. 3 and further validated through statistical testing (see **Table S1** in the supplementary materials), illustrates the significantly reduced ***Heart*** $ \Rightarrow $***Brain, Brain*** $ \Rightarrow $***Heart*** and ***Lungs*** $ \Rightarrow $***Brain*** interactions (Fig. 4 (a)) and elevated interactions from ***Brain*** $ \Rightarrow $***Lungs*** and ***Heart*** $ \Rightarrow $***Lungs*** (Fig. 4(b) in KO animals compared to WT animals. The interaction from the ***Lungs*** $ \Rightarrow $***Heart*** was not found statistically different between the KO and WT animals due to the small number of ssGPDC values of this interaction, and is not depicted in Fig. 4.

### B. Dynamics of Brain-Heart-Lungs Interactions in the Presence of Seizures in SUDEP-Prone Mice

Two out of the eight KO mice (Kv1.1 966 and Kv1.1 967) experienced seizures during their respective 5.5-hour and 7.5-hour recordings of concurrent EEG, ECG and Pleth. We included the first 4-h portion of the recordings from both Kv1.1 966 and Kv1.1 967 in our 4-h analysis of the previous Section II-A. Seizures are typically well-identified electrophysiological events with or without manifestation of clinical symptoms and they can thus constitute referential points to a plurality of investigations and questions ranging from ictogenesis (how seizures form) to epileptogenesis (causes of epilepsy). Within the framework of the present study, we addressed two questions relevant to ictogenesis and seizure occurrence: **(i)** how does seizure occurrence affect the differences in the inter-organ interactions between WT and SUDEP-prone animals, and **(ii)** how do inter-organ interactions in the KO animals change over time in the presence of seizures.

**Fig. 5.**
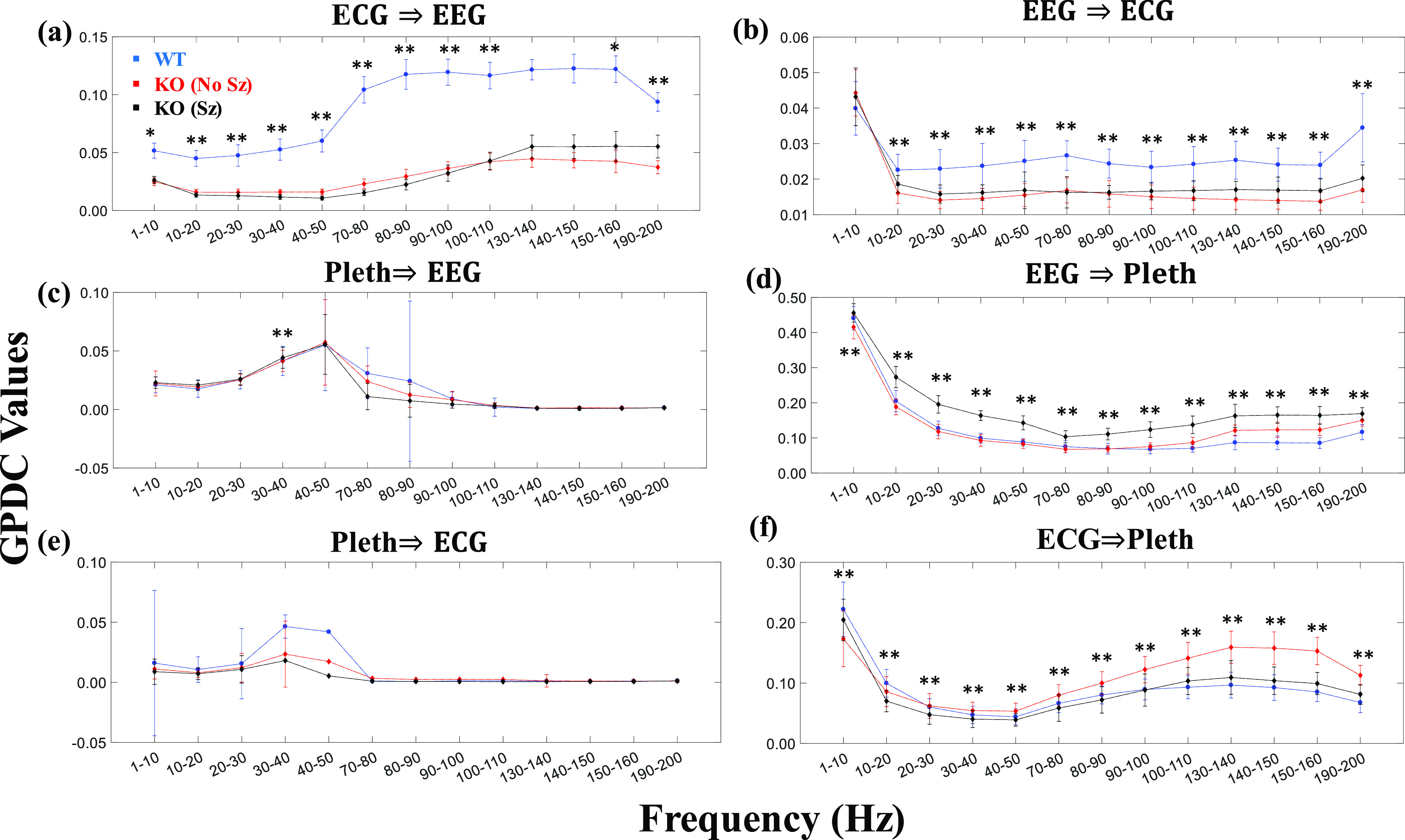
Mean  ± SEM of ssGPDC values of the statistically significant directional connectivities (P < 0.05) between organs over the 4-h EEG-ECG-Pleth recordings in the frequency domain (10 Hz consecutive bands from 1 to 200 Hz excluding the bands with harmonics of 60 Hz power noise) for the WT (blue), SUDEP-prone KO with no seizures (red) and SUDEP-prone KO with seizures (black) mice. (a) Heart ⇒ Brain, (b) Brain ⇒ Heart, (c) Lungs ⇒ Brain, (d) Brain ⇒ Lungs; (e) Lungs ⇒ Heart and (f) Heart ⇒ Lungs. Interactions for which the differences between their median values across KO animals with seizures and those without seizures are statistically not equal as estimated with the non-parametric Mann-Whitney-Wilcoxon rank sum test are indicated by (*) for P-value <0.05, and by (**) for P-value < 0.001.

To address question **(i)** above, we repeated the analysis described in Section III-A for Kv1.1 966 and Kv1.1 967 separately from the rest of the KO mice as these were the only ones with registered seizures during the period of the recording. This resulted in two sets of ssGPDC profiles of the six inter-organ interactions for the two seizing KO mice that we then compared with the ones from the six other non-seizing KO mice (Figure 5). From Fig. 5, and between KO animals with (black lines) or without (red lines) seizures, the most apparently different inter-organ interactions are the ***Brain* *⇒* *Lungs*** (EEG ***⇒*** Pleth) and ***Heart* *⇒* *Lungs*** (ECG ***⇒*** Pleth) interactions. This implies that the presence or absence of seizures does not change significantly the pre-existing differences in those interactions in the KO animals. In particular, the ***Heart* *⇒* *Lungs*** interaction (Fig. 5(f)) in seizing KO animals closely resembles the WT profile across the whole frequency spectrum, whereas the ***Brain⇒* *Lungs*** (Fig. 5(d)) interaction appears elevated in seizing KO mice compared to both non-seizing KO mice and WT controls across the whole frequency spectrum. We can thus conclude that, in this animal model of SUDEP, the communication from the Brain to Lungs becomes abnormally elevated in the presence of seizure activity. However, there are actually two more interactions that are significantly (p < 0.001) affected by the presence of seizures across frequencies: the interactions between the Brain and Heart in both directions (see supplementary Table **S3**).

To address question **(ii)** above we analyzed separately the dynamics of the inter-organ interactions over time (before, during and after seizures) and frequency. For this analysis, the inter-organ interactions that exhibited a) a high percentage of ssGPDC values (see Supplementary Material **Table S4**) and b) statistically significant differences (P < 0.001; Wilcoxon test) between the WT and KO groups (see Fig. 3) across the full frequency spectrum were selected. Thus, only the bi-directional interactions between the brain and heart (***Brain*** $ \Leftrightarrow $***Heart)***, and the efferent interactions from the heart and brain to the lungs (***Brain*** $ \Rightarrow $***Lungs*** and ***Heart*** $ \Rightarrow $***Lungs)*** were considered for dynamical analysis. To maximize the significance of dynamical trends we were attempting to capture, we considered the connectivities in the frequency band for which there was the maximum difference in the respective ssGPDC profiles between the WT and KO groups, that is, the frequency band with the minimum P-value for the Wilcoxon rank sum test on equality of medians.

**Fig. 6.**
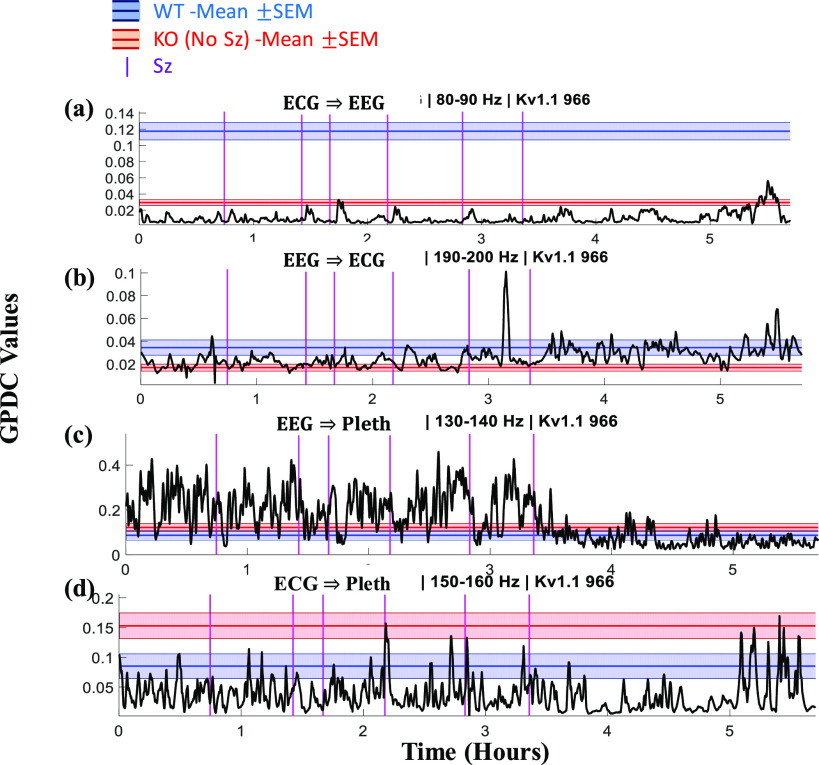
Directional connectivities (smoothed ssGPDC values– see text for details) between brain, heart and lungs over time, peri-ictally and during recovery from seizures, estimated within selected frequency bands from the Kv1.1 966 KO mouse. The 5.5-hour session of EEG, ECG and Pleth in this mouse captured a cluster of six seizures with a subsequent recovery period of 2 hours. (a) Heart $ \Rightarrow $Brain (80-90 Hz), (b) Brain $ \Rightarrow $ Heart (190-200 Hz), (c) Brain $ \Rightarrow $ Lungs (130-140 Hz), (d) Heart $ \Rightarrow $ Lungs (150-160 Hz). Occurrence of seizures is indicated by vertical (Magenta) lines; seizures last for 80 seconds on average. Horizontal bands represent mean ± SEM of ssGPDC values for each interaction and respective frequency band from the WT animals (Blue zone), and the KO animals that did not exhibit seizures during their recording (Red zone).

**Fig. 7.**
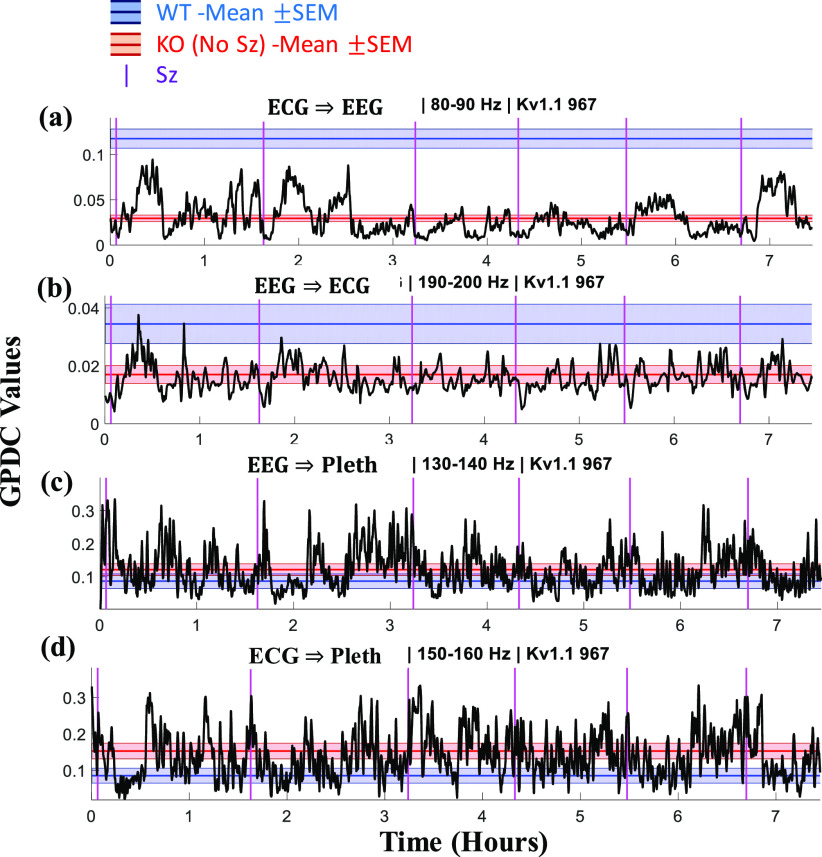
Directional connectivity between brain, heart and lungs peri-ictally within selected frequency bands from A966 KO mouse (smoothed ssGPDC values– see text for details). The 7.5 hour session of EEG, ECG and Pleth in this mouse recorded continuous seizure activity without sustainable recovery. Panels and annotations as in Figure 6.

The smoothed ssGPDC values for these interactions over time for the two KO mice (A966 and A967) in the presence of seizures are shown in Figure 6 and Figure 7 respectively, at the frequency band that each selected interaction exhibited the most statistically significant difference between KO mice and WT mice. Each smoothed ssGPDC value was produced every 10 s by averaging 9 (previous, current, and proceeding) original ssGPDC values over time. Hence, since each original ssGPDC value is estimated from a 10 s window, each portrayed smoothed ssGPDC value in Fig. 5 and Fig. 6 reflects information from a total of 90 s of raw data.

In both Figures 6 and 7, we include two horizontal bands per interaction; each horizontal band (HB) is formed from the mean ± SEM values of the ssGPDC values estimated at the respective frequency sub-band from all WT animals (HB in **blue color**) and all KO animals (HB in **red color**) that had no seizures during their 4-h recordings. In Kv1.1 966 (Fig. 6), six seizures occurred within the first 3.5 hours and no seizures for 2 hours thereafter till the end of the recording. The ***Heart*** $ \Rightarrow $***Brain*** interaction (frequency sub-band: *80-90 Hz)* in this KO mouse was severely impaired during the period with seizures (ssGPDC values well below the HB of WT animals as expected from the results illustrated in Fig. 5, and even below the HB band of the six KO animals that manifested no seizures during their recordings). Increases in strength of this interaction towards normalcy in the post-ictal period of each seizure is noted, as well as a long-term trend (over 2 hours) of gradual increase (recovery) of this connectivity is observed after the end of all seizures (Fig. 6(a)). The ***Brain*** $ \Rightarrow $***Heart*** interaction (frequency sub-band: *130-140 Hz)* is also impaired in the same way as the ***Heart*** $ \Rightarrow $***Brain*** interaction (decreased; below the blue HB but well within the red HB). It is noteworthy that ***Brain*** $ \Rightarrow $***Heart*** recovers much faster than the ***Heart*** $ \Rightarrow $***Brain*** interaction after the end of the seizures, with a trend towards recovery starting even after the fifth seizure in this cluster of 6 seizures, indicating an early recovery of central cardiac control (Fig. 6(b)). The controlling influence of the lungs by the brain (afferent pathways to the lungs) was higher during pre-ictal periods and was reduced to more normal levels after the end of the seizures. In particular, the ***Brain*** $ \Rightarrow $***Lungs*** connectivity (frequency sub-band: *130-140 Hz)* was abnormally high (even beyond the red HB) during pre-ictal periods, and decreases to values within the blue HB for a short time following each seizure's end and permanently after the last seizure (Fig. 6(c)), while the ***Heart*** $ \Rightarrow $***Lungs*** connectivity (frequency sub-band: *150-160 Hz)* is lower peri-ictally throughout the recording compared to the other KO and WT animals (Fig. 6(d)).

The ssGPDC values over time per interaction and frequency for the second KO animal (Kv1.1 967) with seizures during the recording session are given in Figure 7**.** The recording in this animal lasted for 7.5 hours and exhibited frequent seizure activity without a period of long recovery from seizures. Generally, the results shown in Fig. 7 are in agreement with the results reported in the first portion of Fig. 6, that is, during the period that Kv1.1 966 was experiencing seizure activity.

## IV. Discussion

The present study of the brain, heart and lungs in an animal model of SUDEP produced several novel results and insights in the relation of epilepsy and organomics, the emerging science of interactions between organs, by adopting a holistic systems analytical approach. Analysis of directional connectivities between the brain, heart, and lungs revealed significant abnormalities in ***Heart*** $\Rightarrow$ ***Lungs, Brain*** $\Rightarrow$ ***Lungs*** and ***Brain⇔* *Heart*** interactions in SUDEP-prone KO mice versus WT mice (Fig. 5). Specifically, at a highly significant statistical level (p < 0.001), the ***Brain*** $\Rightarrow $ ***Lungs*** and ***Heart*** $\Rightarrow$ ***Lungs*** interactions were higher and ***Brain⇔* *Heart*** interactions were lower in KO mice compared to the corresponding ones from WT mice. The difference in the ***Brain*** $\Rightarrow$ ***Lungs*** interaction was more prominent upon seizure occurrences in KO mice (Fig. 5). Therefore, one hypothesis to be further pursued is if the observed elevated flows of information from the brain and heart to the lungs in the SUDEP-prone KO animals acts as a compensatory mechanism for the decreased bi-directional flow of information between the brain and the heart (Fig. 4).

The directional connectivities between the brain, heart and lungs were found to be frequency-dependent at different degrees. The ***Brain⇔* *Heart*** interactions exhibited a statistically significant decrease in functional connectivity in the KO animals across a wide range of frequencies, while the ***Brain*** $\Rightarrow$ ***Lungs*** interaction was consistently elevated for frequencies above 100 Hz (Fig. 2). High frequencies in the ECG for detection of ventricular tachycardia, as well as in respiration signals for assessment of respiratory abnormalities, have been reported in the past [65]–[70]. However, to our knowledge, this is the first time that we have statistically significant evidence of communication between the brain, heart and lungs in the high frequency portion of the spectrum (>80 Hz). Differences in the efferent pathways from the lungs to heart and brain (***Lungs*** $ \Rightarrow $ ***Brain*** and ***Lungs*** $ \Rightarrow $ ***Heart)*** did not exhibit highly statistically significant differences between the KO and WT animals except for a very narrow frequency band around 80 and 40 Hz respectively (Fig. 2). Respiratory feedback to the brain is regulated by oxygen levels, mechanoreceptor signal transmission and chemical signaling through pH and hormonal activation, and provides lower frequency feedback information to the brain and heart than the fast neurochemical signaling coming from the brain and vagus nerve [71]. The ECG is capable of capturing more minuscule and fast changes in the electromagnetic activity of the cardiac tissue, often under influence by many branches of the vagus nerve extending into the thoracic cavity, the nature of which could explain the large frequency distribution of statistically significant impairments in ***Brain⇔* *Heart*** connectivity in KO animals [10], [11], [67], [68], [72]. The impairment (decrease) in this connectivity in the high frequency spectrum in KO versus WT animals suggests lapses in functional connection from the motor command centers of the brain down to the heart in high frequencies. The activity in high frequencies we measured was not recorded from the traditional cardiac control centers in the brain such as the brainstem. However, activity in other parts of the brain, such as the frontal lobes, as well as the temporal lobes that we recorded from in this study, can modulate cardiac activity through neuro-electrical and endocrine influences [15], [73].

From a dynamics perspective, we studied the inter-organ interactions with respect to the timing of events that were very well-defined, the epileptic seizures, and compared their pre- and post-ictal values to referential values of inter-organ interactions during seizure-free periods from WT and SUDEP-prone KO animals. The general pre-ictal trend was an elevated input to the lungs from the brain and a reduced input to the heart from the brain; post-ictally (after seizure end), the opposite changes (i.e., decreases) were observed in those interactions. These changes were most prominent at high frequencies. Furthermore, the most distinct dynamics occurred over larger timescales (2 hours), that is, when comparing the period with intense seizure activity to the subsequent seizure-free (recovery) period (Fig. 6). These findings point to at least three complementary directions for future investigations into: **a) *resetting of dynamics by seizures*** and/or seizure clusters (mostly displayed in this study by the ***Brain*** $ \Rightarrow $***Lungs*** and ***Brain*** $ \Rightarrow $***Heart*** interactions), a concept that we postulated and have shown in the past by spatiotemporal analysis of scalp and intracranial EEG from patients and epileptic rats [38], [41], [43], [49], [54], [74]–[76], **b)** the potential use of the dynamics of specific interactions (e.g., the ***Brain*** $ \Rightarrow $***Lungs*** interaction) for ***prediction of seizures***; studies have shown increased inflammatory processes in the larynx in cases of the similarly infrequent and sudden condition, the Sudden Infant Death Syndrome (SIDS), suggesting the involvement of abnormal hyper-reactivity of the reflexes and apnea [77]. Such increased reactivity of laryngeal reflexes is hypothesized to be present in some cases of SUDEP [78], which may be reflected and potentially quantified by our directed network analysis in this study between plethysmography and EEG, **c)** identifying periods of ***seizure susceptibility*** (e.g., by the ***Lungs*** $ \Rightarrow $***Brain*** and/or ***Lungs*** $ \Rightarrow $***Heart*** interaction) [74], [79]–[84].

To our knowledge, this study is the first attempt to assess the communication between the brain, heart and lungs together in controlled experiments centered on a neurodegenerative disease. This study is also a rare comparison of control and diseased groups in epilepsy, as typical studies investigating mechanisms of epilepsy and SUDEP lack control subjects due to the nature of human data collection in clinical epilepsy research. Even though important and statistically very significant results were generated through our analysis of the experimental data, we would like to acknowledge the following experimental and theoretical limitations of the present study that could guide future studies. **First,** the interactions we investigated showed frequency dependence, especially interactions that included the respiratory system. Such intricate frequency dependencies may be better quantified via a cross-frequency analysis [85]–[87] since cross-frequency interactions are not well captured by using the linear MVAR modelling and coherence approach, because this approach theoretically can capture and quantify activities across organs at the same frequency in the spectrum. Frequency nesting, where the amplitude at one frequency (often high frequency) in one signal (organ) depends on the amplitude at a different frequency (usually low frequency) of another signal (organ), is an example of cross-frequency interactions. Cross-frequency phase locking, cross-frequency coherence, and stochastic measures of cross-frequency dependence also exist [88]. However, such measures may require large amounts of data in order to produce statistically significant results, whereas linear modelling is computationally fast and can work with short segments in order to deal with non-stationarities. **Second,** in theory, linear modelling can accurately model only linear systems. However, linear models can approximate nonlinear systems and nonlinear behavior under specific constraints (e.g., operating region / spatio-temporal state). In the case of a biological signal, where the underlying system is typically nonlinear and the constraints are relatively unknown, the linear modelling approach should always constitute a first approximation that could lead to learning of the constraints for a subsequent better linear approximation and so on [89]–[91]. **Third,** despite the experiments being state-of-the-art with the appropriate controls, it remains to be seen if our results are general to SUDEP or specific only to the *Kcna1* KO animal model of SUDEP.

## V. Conclusion

In this study, we employed directed network connectivity analysis between the brain, heart and lungs on a rare dataset of concurrently recorded EEG, ECG and Pleth signals in WT and SUDEP-prone KO animals. The results of this network analysis suggest important frequency-dependent abnormalities in the neuro-cardio-respiratory system of KO mice. Connections between the brain and heart, and the brain and lungs showed the most statistically significant abnormalities in the SUDEP-prone KO animals. Further analysis of their dynamics showed that they may play an important role in the generation of seizures (ictogenesis). Pre-ictal elevation of ***Brain*** $ \Rightarrow $***Lungs*** efferent connections appears to be a key feature preceding seizure manifestation, implying hyper-reactivity in this branch of the system involving either the respiratory sensory centers in the brain, the pathway between the lungs and the brain, or the sensory system in the lungs. In addition, decreased ***Heart⇔* *Brain*** activity seems to also play a key role in the manifestation of seizures and possibly SUDEP. These novel findings, following further validation in additional datasets from animals and humans, may be incorporated in seizure detection and prediction algorithms within intelligent neuromodulation devices for seizure control and for detection of periods of susceptibility to seizures and risk of SUDEP.

## Supplementary Materials

In the supplementary material, the estimation of directed connectivities by MVAR and the assessment of their statistical significance are briefly described. The percentage of GPDC values rendered not statistically significant (p>0.05) per interaction and frequency band for each of the two genotypes (KO and WT) is illustrated in **Figure S1.** The results from our analysis of the impact of the order of the MVAR model on the values of inter-organ connectivities are given in **Figure S2.** In **Tables S1, S3** and **S4,** using non-parametric statistical tests, the p-values of the differences in the medians of connectivity per interaction, respectively, between: **1)** WT (all animals) and KO (all animals) averaged across all frequencies (as depicted in Fig. 3), **2)** KO (only animals without seizures) and KO (only animals with seizures) at each individual frequency band for validation of conclusions from Fig. 5, and **3)** WT (all animals) and KO (only animals with seizures) at each individual frequency band in order to select the frequency bands for the dynamic analysis depicted in Fig. 6 and Fig. 7. **Table S2** depicts p-values for each inter-organ interaction with each of the rest of the other inter-organ interactions per animal genotype (WT and KO).


