# Supplementary material for: Directed Connectivity Analysis of the Neuro-Cardio- and Respiratory Systems Reveals Novel Biomarkers of Susceptibility to SUDEP
Source: IEEE Open J Eng Med Biol. 2020 Nov 6;1:301–11. doi: 10.1109/OJEMB.2020.3036544 (PMC8249082; doi:10.1109/OJEMB.2020.3036544)
Supplement: Supplementary file 1 [file supp1-3036544.pdf]

Supplementary Materials

Directed Connectivity Analysis of the Neuro-Cardio-Respiratory Systems Reveals Novel Biomarkers of Susceptibility to SUDEP

T. Noah Hutson*, *Member* IEEE, Farnaz Rezaei, Nicole M. Gautier, Jagadeeswaran Indumathy, Edward Glasscock, Leonidas Iasemidis, *Fellow* IEEE

SI. Connectivity Analysis of EEG-ECG-Pleth signals

For each set of 10-s running windows *w*(*t*) on the signals extracted at the same time from all 6 channels (4 EEG, 1 ECG, 1 Pleth), the MVAR model linearly fits the column vectors *X*(*t*) which are of dimension *D* (*D*=6) and have components the values of the 6 recorded signals at time *t*, for every *t* within the window *w*(*t*). That is:

$$X(t) = \sum_{\tau=1}^{p} \boldsymbol{A}(\tau)X(t-\tau) + E(t) \tag{1}$$

where the time index *t* is from 1 to *N*, with *N* being the number of data points within a time window (*N*=5000) per time series, *p* is the order of the model (*p*=7), and τ is increasing in steps of the time delay between samples (we used τ=1, that is, in time units, equal to the sampling period of 1 / (500Hz) = 2 ms). Matrices $\boldsymbol{A}(\tau)$ have the model's coefficients; the fitting error values are the components of the vector *E*(*t*) (in the ideal MVAR model fit, *E*(*t*) is multivariate Gaussian white noise). The coefficients of the MVAR model were estimated via the Vieira-Morf partial correlation method [91]. Taking the discrete Fourier Transform of both sides of (1) and rearranging, we have: $\left[I - \sum_{\tau=1}^{p} \boldsymbol{A}(\tau)e^{-i2\pi f\tau}\right] \cdot X(f) = E(f)$, where *I* is the identity matrix. Then, by defining:

$$\boldsymbol{B}(f) = I - \sum_{\tau=1}^{p} \boldsymbol{A}(\tau)e^{-i2\pi f\tau} \tag{2}$$

Under the assumption of strict causality (the *D*×*D* covariance matrix $\boldsymbol{S} = \left[\sigma_{ij}\right]_{i,j=1,\dots,D}$ of $E(t)$ is diagonal), GPDC is then derived by decomposing the partial coherence applying spectral factorization theorem [34]. GPDC from site *j* to site *i* at frequency *f* is defined as:

$$GPDC_{j\to i}(f) = \frac{\frac{\left|\boldsymbol{B}_{ij}(f)\right|}{\sigma_{ii}}}{\sqrt{\sum_{k=1}^{D} \frac{\left|\boldsymbol{B}_{kj}(f)\right|^2}{\sigma_{kk}^2}}} \tag{3}$$

where $\boldsymbol{B}_{ij}(f)$ is the $(i,j)^{\text{th}}$ element of the matrix $\boldsymbol{B}(f)$ that denotes the interaction from *j*→*i*, σ_{ii} are the diagonal elements of $\boldsymbol{S}$. According to (3), GPDC is defined by normalizing by the "power" of each receiving node (time series).

The statistical significance of the GPDC values for each interaction derived from each 10-s window was then determined. The statistical criteria for inferring the statistical significance and confidence interval of the derived frequency-domain Granger causality-based connectivity measures are recent and have been discussed by a small number of researchers. Monte Carlo simulations was the initial technique that was developed to assess the statistics of the connectivity measures [92]. Surrogate analysis was used as the first unbiased statistical assessment by Kaminiski *et al.* in 2001 [93], [94]. In 2006, Baccala *et al.* compared the surrogate method to prediction error-based methods and concluded that they are essentially equivalent in most cases [95]. Schelter *et al.* introduced the asymptotic behavior for PDC in 2006 [96]. More recently, Toppi *et al.* compared the surrogate analysis (also denoted as causal Fourier transform shuffling (CFT) [97] with the introduced state-of-the-art unified asymptotic analysis by Baccala *et al.* and concluded that they are mainly identical [98]. In this study, we have followed this asymptotic analysis for evaluation of the connectivity measures from the MVAR modeling of our data.

The significance of the connectivity measure $\text{GPDC}_{i,j}(f)$ at a specific frequency *f* between two nodes (channels) *i* and *j* was tested according to the following null hypothesis:

$$H_0 : \left|\text{GPDC}_{i,j}(f)\right|^2 = 0;\ \forall i,j \in \{1,\dots,D\} \tag{5}$$

Rejecting the H_0 at a specified α significance level provides a strong conclusion for existence of a significant connectivity. Confidence interval for the existing connections was estimated by determining the asymptotic distribution of the GPDC measures according to [95]. Then, to reject non-statistically significant GPDC values, we used the statistical significance level of α=0.05. Only the thus identified significant interactions were further analyzed in this study.

For all animals, statistically significant GPDC values for each of the three organs (brain, heart and lungs) were averaged within 10Hz consecutive and non-overlapping frequency bands per 10s window of data. Finally, the thus estimated 6 directed paired interactions (***Heart*** **⇒** ***Lungs, Lungs*** **⇒** ***Heart, Heart*** **⇒** ***Brain, Brain*** **⇒** ***Heart, Lungs*** **⇒** ***Brain*** *and* ***Brain*** **⇒** ***Lungs***) every 10 s per animal and frequency band, were also aggregated to account for the difference in the number of recording channels per organ. For example, in the aggregation of the ***Heart*** **⇒** ***Brain*** interaction, the statistically significant GPDC values per frequency band from ECG to each of the 4 EEG sites were averaged. These averaged GPDC values for each interaction and frequency band were then grouped into each of the two genotypes (WT and KO). Thus, for 7 animals in the WT group and 8 in the KO group, with a 4-hour recording per animal and GPDC values being estimated every 10 s, there were a total of 10,080 GPDC values per interaction and frequency

band for the WT group, and 11,520 GPDC values per interaction and frequency band for KO group of animals. Thus, due to the availability of such big sample sizes, we are having high statistical power and hence a higher probability for existing statistically significant differences per interaction and frequency band across the two groups to be detected.

SII. Statistical analysis of results

A. Neuro-Cardio-Respiratory Connectivity in WT and KO animals

In **Figure S1** we illustrate the importance of considering the statistical significance of each interaction. In particular, we plot the percentage (PER) of non-statistically significant GPDC values per interaction, genotype and frequency bin by using a

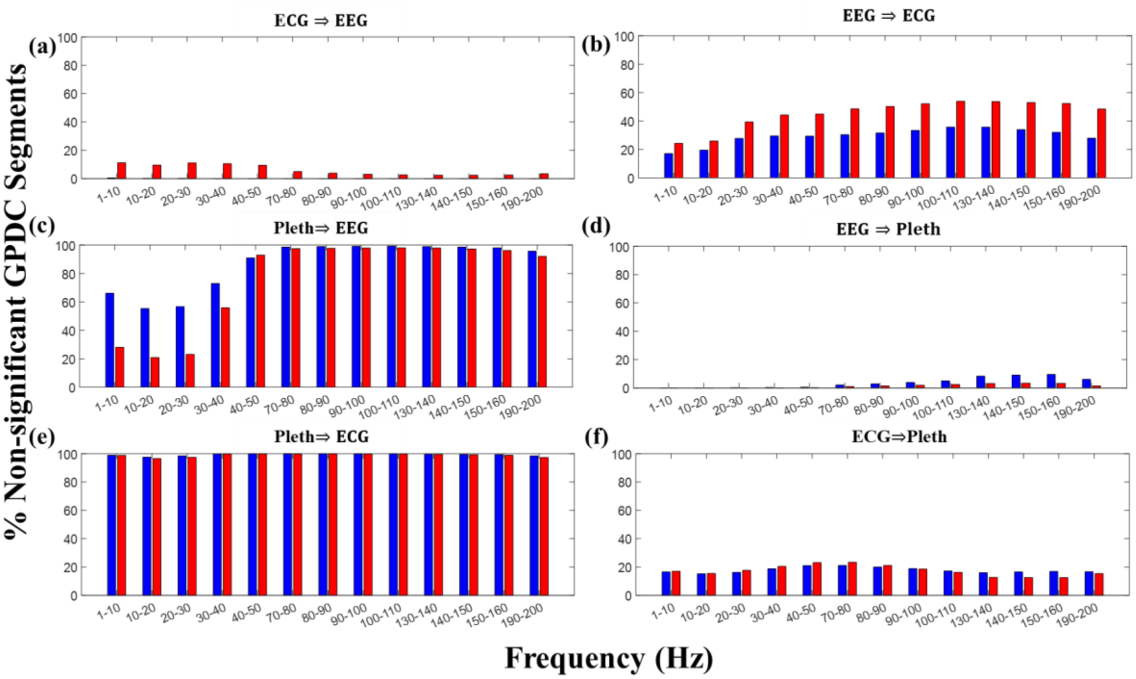

Figure S1. Percentage (PER) of GPDC values rendered not statistically significant (p>0.05) per interaction and frequency band for each of the two genotypes (KO and WT). The employed asymptotic significance test is applied to each GPDC value generated per interaction from the fitting by MVAR of data within each 10s running window. The PER values in red correspond to the KO and in blue to the WT genotype. Each of the panels shows the PER from one of the six different inter-organ interactions: **(a)** ***Heart ⇒ Brain*****,** **(b)** ***Brain ⇒ Heart*****;** **(c)** ***Lungs ⇒ Brain*****,** **(d)** ***Brain ⇒ Lungs*****;** **(e)** ***Lungs ⇒ Heart*** and **(f)** ***Heart ⇒ Lungs*****.**

Table S1

P-values (Wilcoxon rank sum test) of three Null Hypotheses (H_0) about the difference of Median values of Brain-Heart-Lungs directional GPDC interactions between WT and KO animals over the full frequency spectrum.

	Interaction					
H_0	**ECG->EEG**	**EEG->ECG**	**Pleth->EEG**	**EEG->Pleth**	**Pleth->ECG**	**ECG->Pleth**
**KO Exhibits Equal Median of Connectivity**	<<0.001	<0.001	<0.001	<0.001	0.1282	<0.001
**KO Exhibits Decreased Median of Connectivity**	<<0.001	<0.001	0.9998	1	0.0641	1
**KO Exhibits Increased Median of Connectivity**	1	1	<0.001	<0.001	0.9359	<0.001

IEEE Open Journal of Engineering in Medicine and Biology

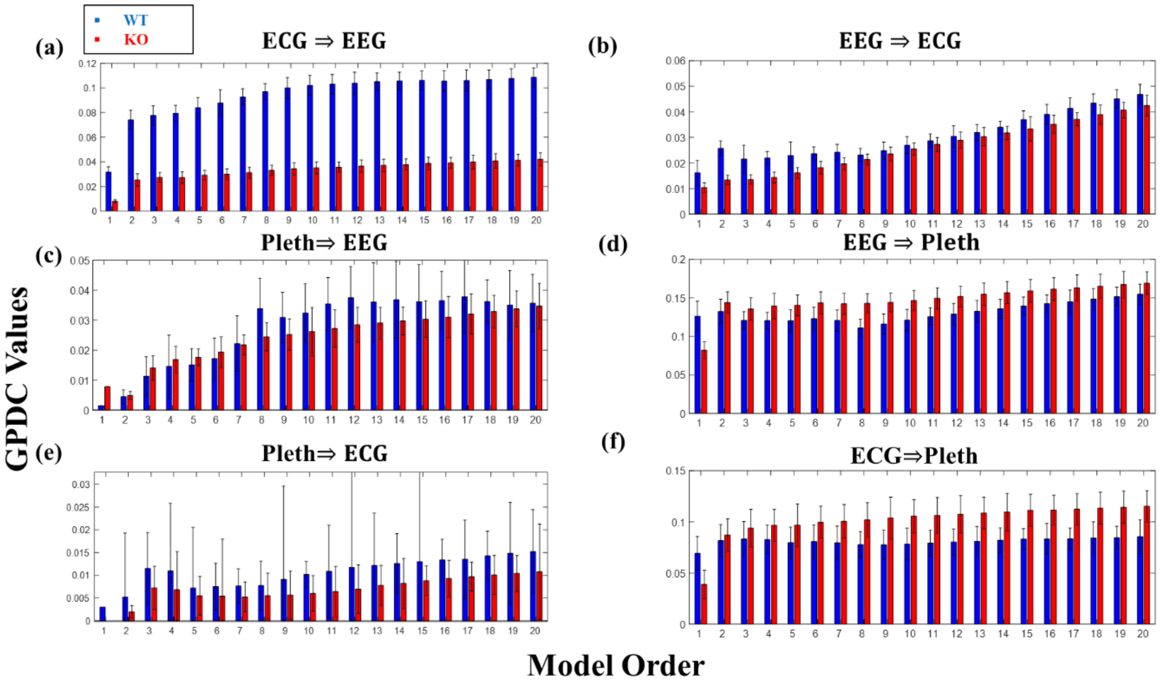

Figure S2. The six inter-organ statistically significant GPDC values (mean ± SEM; estimated every 10 s and averaged over the full frequency spectrum of 1-200 Hz and the full 4-hour epoch) as a function of the order p (from 1 to 20) of the employed MVAR models (red bars for KO and blue bars for WT animals).

statistical significance level of α=0.05. The interactions with a high percentage of statistically insignificant GPDC values are the ones from the ***Lungs to Brain*** (for f > 40 Hz) ***and Lungs to Heart*** (Figure 2(c) and (e) respectively). From the rest of panels in Figure 2 (a, b, d, f), we observe that the interactions with the greatest number of ssGPDC values (interactions that are statistically validated) are the following in descending order: [***Heart⇒Brain]*** ≚ ***[Brain⇒Lungs]*** > [***Heart⇒Lungs]*** > ***[Brain⇒Heart].*** Although a dependency of PER on the frequency and genotype is observed for each interaction, the above order of significance of interactions was relatively consistent across genotypes and a wide range of frequencies as is evident from Figure 2.

Table S1 depicts the P-values from testing three null hypotheses (+, -, =) about for the respective differences in each inter-organ interaction based on three non-parametric Wilcoxon rank sum tests. For example, in the case of ***Brain⇒Heart*** interaction, we see that the null hypotheses of the "KO animals having equal median value of connectivity to that of WT", as well as that "KO animals have higher median value of connectivity than that of WT" are rejected with P<0.001; that is, the medians in this interaction are statistically not equal and the KO animals have lower median value of connectivity than that of WT (in line with the test of the 3rd null hypothesis of "KO animals having lower median value of connectivity than that of WT", which cannot be rejected (P=1)).

Table S2 depicts the p-values from testing the null hypotheses that each row element (interaction i) exhibits a lower median value of connectivity than each corresponding column element (interaction j). This table depicts the results separately for WT (in (a)) and KO (in (b)) genotypes. For example, in Table S2(a), row 1 column 2, the p-value is <0.001, therefore the null hypothesis that ***Brain⇒Lungs*** exhibits a lower median of connectivity than ***Heart⇒Lungs*** in wild type animals is rejected.

Finally, the aforementioned differences in the inter-organ interactions between WT and KO animals are observed for a large range of MVAR model orders. **Figure S2** presents the respective results to the ones in Figure 3 after the whole analysis was repeated on the same data with MVAR models of p=1 to 20 orders. Results different from the ones in Fig. 3 were found only with models of low order (p=1 and 2) in the ***Brain⇒Lungs*** and ***Heart⇒Lungs*** interactions. The ***Lungs⇒Brain*** and ***Lungs⇒Heart*** **interactions** exhibited the largest standard deviations of the mean for a wide range of p. From p=5 and all the way up to p=20, results of the differences of the inter-organ interactions between the two genotypes are of the same sign, consistent with Figure 3: the ***Heart⇒Brain, Brain⇒Heart, Lungs⇒Brain*** and ***Lungs⇒Heart*** interactions in the KO animals are reduced, while the efferent interactions are elevated from ***Brain⇒Lungs*** and ***Heart⇒Lungs***. This investigation implies robustness of the results in this study from MVAR models of order p > 5.

B. Dynamics of brain-heart-lungs interactions in the presence of seizures in SUDEP-prone mice

To further validate our visual observations from Figure 5 in the main manuscript, we performed a Wilcoxon-rank-sum test between the KO animals with seizures and the KO animals that did not exhibit seizures during our recordings. The corresponding P-values per frequency band and interaction are

shown in **Table S4**. To maximize the statistical significance of the observed dynamic trends in connectivities, we re-estimated the connectivities in the frequency band for which there was the maximum difference in the respective ssGPDC profiles between the WT and KO groups (frequency band with the minimum P-value for the Wilcoxon rank sum test on equality of medians – see **Table S4**). The thus derived ssGPDC values per determined frequency band for each interaction and genotype we plotted over time in Figures 6 and 7.

Table S2

P-values estimated via Wilcoxon-Rank-Sum tests of the null hypotheses that each row (i) element (interaction) exhibits a lower median value of connectivity than the corresponding column (j) element (interaction) for the (a) WT animals and (b) KO animals. Non-statistically significant differences per interaction (Wilcoxon rank sum test; p>0.05) are shaded in gray.

Interaction in WT animals

(a)

	**EEG->Pleth**	**ECG->Pleth**	**ECG->EEG**	**EEG->ECG**	**Pleth->ECG**	**Pleth->EEG**
**EEG->Pleth**		<0.001	<0.001	<0.001	<0.001	<0.001
**ECG->Pleth**	1		1	<0.001	<0.001	<0.001
**ECG->EEG**	1	<0.001		<0.001	<0.001	<0.001
**EEG->ECG**	1	1	1		<0.001	<0.001
**Pleth->ECG**	1	1	1	1		1
**Pleth->EEG**	1	1	1	1	<0.001	

Interaction in KO animals

(b)

	**EEG->Pleth**	**ECG->Pleth**	**ECG->EEG**	**EEG->ECG**	**Pleth->ECG**	**Pleth->EEG**
**EEG->Pleth**		<0.001	<0.001	<0.001	<0.001	<0.001
**ECG->Pleth**	1		<0.001	<0.001	<0.001	<0.001
**ECG->EEG**	1	1		<0.001	<0.001	<0.001
**EEG->ECG**	1	1	1		<0.001	<0.001
**Pleth->ECG**	1	1	1	1		1
**Pleth->EEG**	1	1	1	1	<0.001	

Table S3

P-values (Wilcoxon rank sum test) per interaction and frequency band from testing the Null Hypothesis H_0 that “Brain-Heart-Lungs directional interactions between SUDEP-prone animals with seizures and SUDEP-prone animals without seizures are equal”.

Frequency (Hz)

Interaction	1-10	10-20	20-30	30-40	40-50	70-80	80-90	90-100	100-110	130-140	140-150	150-160	190-200
**ECG->EEG**	<0.001	<0.001	<0.001	<0.001	<0.001	<0.001	<0.001	<0.001	<0.001	<0.001	<0.001	<0.001	<0.001
**EEG->ECG**	<0.001	0.00295	<0.001	<0.001	<0.001	<0.001	<0.001	<0.001	<0.001	<0.001	<0.001	<0.001	<0.001
**Pleth->EEG**	<0.001	<0.001	1.04E-06	<0.001	0.0812	<0.001	0.0357	0.2349	0.0016	0.9885	0.4096	0.2151	0.2413
**EEG->Pleth**	<0.001	3.66E-01	2.25E-10	<0.001	<0.001	<0.001	<0.001	<0.001	<0.001	<0.001	<0.001	<0.001	<0.001
**ECG->Pleth**	<0.001	<0.001	0.09	<0.001	<0.001	0.308	0.0103	<	<0.001	<0.001	<0.001	<0.001	<0.001
**Pleth->ECG**	0.0078	0.184	0.236	0.079	0.129	0.383	0.693	0.932	0.522	0.912	0.779	0.850	0.864

Table S4

P-values per interaction and frequency band from testing the Null Hypothesis H_0 that “Brain-Heart-Lungs directional interactions between WT and SUDEP-prone animals with seizures are equal”. The frequency band with the lowest P-value for each interaction is indicated in red. Non-statistically significant interactions (Wilcoxon rank sum test; p>0.05) are shaded in gray.

Frequency (Hz)

Interaction	1-10	10-20	20-30	30-40	40-50	70-80	80-90	90-100	100-110	130-140	140-150	150-160	190-200
**ECG->EEG**	<<0.001	<<0.001	<<0.001	<<0.001	<<0.001	<<0.001	**<<0.001**	<<0.001	<<0.001	<<0.001	<<0.001	<<0.001	<<0.001
**EEG->ECG**	<0.001	0.0029	<0.001	<0.001	<0.001	<0.001	<0.001	<0.001	<0.001	<0.001	<0.001	<0.001	**<0.001**
**EEG->Pleth**	<0.001	0.366	<0.001	<0.001	<0.001	<0.001	<0.001	<0.001	<0.001	<<0.001	**<<0.001**	<<0.001	<0.001
**ECG->Pleth**	<0.001	<0.001	<0.001	<0.001	<0.001	<0.001	<0.001	<0.001	<0.001	<0.001	<0.001	**<0.001**	<0.001
